# Enhanced Recombinant Protein Production Under Special Environmental Stress

**DOI:** 10.3389/fmicb.2021.630814

**Published:** 2021-04-15

**Authors:** Xinyi Chen, Chun Li, Hu Liu

**Affiliations:** ^1^Key Laboratory of Medical Molecule Science and Pharmaceutics Engineering, Ministry of Industry and Information Technology, Institute of Biochemical Engineering, School of Chemistry and Chemical Engineering, Beijing Institute of Technology, Beijing, China; ^2^Key Laboratory for Industrial Biocatalysis, Ministry of Education, Department of Chemical Engineering, Tsinghua University, Beijing, China; ^3^Center for Synthetic & Systems Biology, Tsinghua University, Beijing, China

**Keywords:** microorganism, recombinant protein, environmental stress, low temperature, hypoxia, microgravity, osmolality

## Abstract

Regardless of bacteria or eukaryotic microorganism hosts, improving their ability to express heterologous proteins is always a goal worthy of elaborate study. In addition to traditional methods including intracellular synthesis process regulation and extracellular environment optimization, some special or extreme conditions can also be employed to create an enhancing effect on heterologous protein production. In this review, we summarize some extreme environmental factors used for the improvement of heterologous protein expression, including low temperature, hypoxia, microgravity and high osmolality. The applications of these strategies are elaborated with examples of well-documented studies. We also demonstrated the confirmed or hypothetical mechanisms of environment stress affecting the host behaviors. In addition, multi-omics techniques driving the stress-responsive research for construction of efficient microbial cell factories are also prospected at the end.

## Introduction

Production of recombinant proteins is a promising research field in many industries with particular requirements for biochemical active products, such as cosmetic industry, food industry and pharmaceutical industry. Many microorganisms, such as bacteria, yeast, and filamentous fungi are used or engineered as cell factories for the production of foreign proteins. Depending on their characteristics, they are extensively used in various applications.

*Escherichia coli (E. coli)* is the most commonly used prokaryote for the production of heterologous proteins since its physiology and metabolism have been extensively characterized (Rosano and Ceccarelli, 2014). Besides, *E. coli* has unparalleled fast growth kinetics and is able to achieve the high density cultivation easily (Choi et al., 2006; Sezonov et al., 2007). *Bacillus subtilis (B. subtilis)* is a widely used Gram-positive bacteria with an excellent secretion system, which is beneficial for industrial enzyme production (Welsch et al., 2015). Yeasts are very important eukaryotic workhorse microorganism with a great biodiversity existing among them (Buckholz and Gleeson, 1991; Sudbery, 1996; Muller et al., 1998). *Saccharomyces cerevisiae (S. cerevisiae)* has been used for centuries in food production, and is considered as Generally Recognized As Safe (GRAS) microbial host. Oleaginous yeast *Yarrowia lipolytica (Y. lipolytica)* has an excellent lipid accumulation capacity and is generally used as an attractive candidate for microbial oil production (Tai and Stephanopoulos, 2013). Methylotrophic yeasts, including *Komagataella phaffii* (*Pichia pastoris*), *Hansenula polymorpha (H. polimorpha)* and *Candida tropicalis (C. tropicalis)*, have a characteristic pathway with many methanol-oxidizing enzymes demanded to metabolize one-carbon compounds, which endows methylotrophic yeasts with strong and tightly regulated promoters to act as chassis hosts (Cereghino and Cregg, 2000; Cregg et al., 2009; Potvin et al., 2012). As an eukaryotic model microbe, yeasts combine the advantages of high growth rate, complete protein processing capacity [protein folding, secretion, assembly, post-translational modification, disulfide bond formation and proteolytic maturation (Nielsen, 2013)] and relatively easy genetic modification. Another important eukaryotic host candidate is *Filamentous fungi* with extraordinary capacity for extracellular enzyme synthesis and secretion, including *Aspergillus* species, *Trichoderma* species, *Penicillium* species and *Rhizopus* species (Ward, 2012). As reported, the ability of such species to produce some recombinant extracellular proteins can reach up to about one hundred grams per liter (Demain and Vaishnav, 2009). Furthermore, more than 80% of the world’s biotech drugs are extracted from animal cells and the use of mammalian cell factories to produce recombinant proteins is of great significance in pharmaceutical engineering.

**FIGURE 1:**
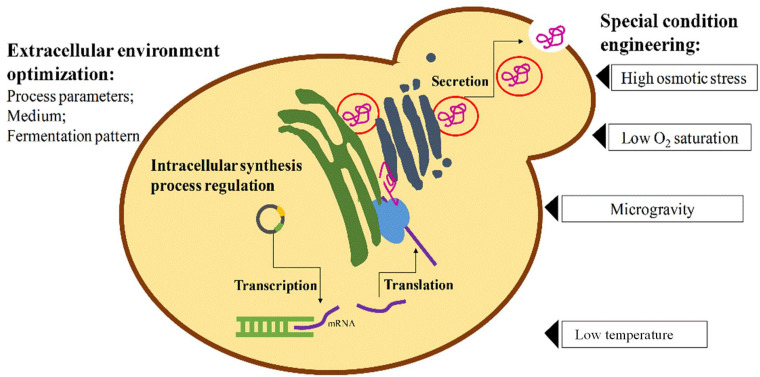
General intracellular and extracellular engineering strategies for enhancing heterologous protein expression in yeasts.

Regardless of the selection of microorganism used for recombinant protein production, the traditional methods used for enhancing protein expression level are parallel, including optimizing foreign protein sequence, selecting suitable promoters, terminators and transcriptional factors, improving translation efficiency, engineering secretion signal sequence or secretory pathway for efficient extracellular protein production (Domínguez et al., 1998; Grabherr et al., 2002; Makino et al., 2011; Dehnavi et al., 2016; Figure 1). In addition, the responses of cells to the external environment can also affect the expression level of microorganism hosts. Therefore, the fermentation parameters such as temperature, pH, cell density, induction time and inducer concentrations are all worthy of comprehensive optimization, so that host cells can maintain a suitable state with high enzyme activity and protein production efficiency (Figure 1). However, in recent years, it was noticed that some special environments or stressful conditions imposed on host organisms could create an unexpected positive effect on their heterologous protein production though some of these phenomena have not been elucidated mechanistically (Figure 1). It was once considered that overexpression of heterologous proteins beyond a certain threshold will become a severe burden on microbial growth and metabolism, leading to lower productivity of target protein. But in fact, under a stressful condition in many cases, the host cellular systems would regulate their metabolic routines to response to the stressful environment, during which some stress response genes in microorganisms would be up- or down-regulated transiently and finally return to the near normal level (Gasch and Werner-Washburne, 2002; Mager and Siderius, 2002; Mattanovich et al., 2004). Within a certain range, such shift of cell adaption can allow microorganisms into a better state with a regulated carbon metabolism, amino acid metabolism, lipid metabolism and even membrane organization, to improve the production of recombinant proteins.

In this review, some stressful conditions including low temperature, hypoxia, microgravity and high osmolality are covered and their beneficial effects on heterologous protein expression in microorganism hosts are discussed extensively. Typical examples of their applications in improving protein production and the potential mechanisms are also summarized. In addition, the applications of the omics techniques in driving the stress-responsive research are discussed and their potential in the construction of efficient microbial cell factory are prospected.

## Environmental Stress for Enhancing Recombinant Protein Production

Although it is recognized that microorganisms should be cultivated in an optimal environment for rapid growth and reproduction, some special environments such as high osmotic pressure, low O_2_ saturation, microgravity and other environmental stimulations can actually create a positive influence on the microbial metabolism. These stressful conditions are able to induce microorganisms to express certain stress-induced genes, change the cellular physiological state or create a new cellular homeostasis, which has been developing into a significant research field.

### Low Temperature

It’s acknowledged that low cultivation temperature can lead to a higher foreign protein production level in microorganism (Jahic et al., 2003; Mattanovich et al., 2004). The advantages of low cultivation temperature include the favorable effects on energy metabolism, protein folding and secretion, protein degradation and aggregation in recombinant microorganism cells (Georgiou and Valax, 1996; Cassland and Jonsson, 1999; Li et al., 2001; Zepeda et al., 2018), moreover, on the economical efficiency in industrial fermentation production. Sometimes, a low cultivation temperature is even a requisite condition for the production of some particular proteins, such as psychrophilic and mesophilic proteins (Vigentini et al., 2006). The directly positive effect of low temperature on recombinant protein production is reflected in the improved yield or activity of the target protein. Dragosits et al. reported a threefold increased specific productivity of an antibody Fab fragment at a lower cultivation temperature in *P. pastoris*. Meanwhile, a reduced flux through the TCA-cycle, reduced levels of proteins response to oxidative stress and reduced cellular levels of molecular chaperones were observed (Dragosits et al., 2009). Li et al. (2001) achieved a dramatically increased yield of antifreeze protein in *P. pastoris* under a low cultivation temperature, which might be the result of increased protein folding and an increased cell viability. Cassland and Jönsson achieved a 16-fold higher laccase activity at 19°C than that obtained at 28°C in *S. cerevisiae*, and similar results were also observed in *P. pastoris* (Cassland and Jonsson, 1999).

The low temperature benefits recombinant protein production mainly at the transcriptional level or translational/post-translational level. At the transcriptional level, the recombinant protein expression at low temperature can be under the transcriptional control of cold-induced promoters. For instance, the promoter of the major *E. coli* cold shock protein, *CspA*, has been employed for the low-temperature induced protein expression. Synthesis of β-galactosidase was efficiently repressed at 37°C but rapidly induced upon transfer to the 15–30°C range, leading to a three- to five- fold increase in specific activity relative to control cultures (Vasina and Baneyx, 1996). However, this promoter became repressed 60–120 min after initiation of cooling. The researchers then used a mutated gene encoding the ribosomal binding factor *RbfA* in host cells and abolished the promoter repression successfully, leading to a high-level expression of β-galactosidase for 7 h after the temperature downshift (Vasina et al., 1998). Afterward, the *cspA* promoter was employed to construct a cold-shock inducible expression system for the expression of some cold-active proteins, psychrophilic enzymes and even toxic mesophilic and thermophilic proteins which are deleterious to the host cell growth (Bjerga and Williamson, 2015; Bjerga et al., 2016). Analogous systems have been developed for *B. subtilis* where the cold-inducible promoter of the desaturase encoding gene (*des*) was used for intra- and extracellular synthesis of recombinant proteins. Production of recombinant proteins started within the first 30 min after temperature downshift to 25°C and continued for about 5 h (Thuy Le and Schumann, 2007). Moreover, this promoter, in combination with a *capB* 5′ UTR regulatory element and the transcription terminator of *B. subtilis* cold-inducible bkd operon caused a higher mRNA stability and thus a further significant increase in protein expression, which has been verified by heterologous production of β-galactosidase from *Pseudoalteromonas haloplanktis* TAE79A, xylanase from *B. subtilis* and α-glucosidase from *S. cerevisiae* (Janiyani and Ray, 2002). A new cold-inducible expression vector with the CnAFP promoter from the polar diatom *Chaetoceros neogracile* was constructed and a promising transcriptional regulatory element responsive to low temperature was developed (Kim et al., 2020). Similar to cold induction, thermally induced gene expression was achieved through pL and/or pR phage lambda promoters regulated by the thermolabile cI857 repressor (Valdez-Cruz et al., 2010). Different genetic elements reported in literature and their applications in recombinant protein production are summarized in Table 1. In addition, the antarctic psychrotrophic bacteria provide useful model systems for studying cold adaptation and cold inducible promoters. The urocanase gene *hutU* from the *Psychrotroph* was suggested to be inducible upon a downshift of temperature (22 to 4°C). A transcription initiation site specific to the cells grown at 4°C and a characteristic CAAAA sequence at the −10 position of the promoters were discovered (Janiyani and Ray, 2002). Duilio et al. characterized a strain named TAC125, which has been isolated from Antarctic seawater and classified as *Pseudoalteromonas haloplanktis (P. haloplanktis)*. The plasmid pMtBL from TAC125 has been the first “cold” genetic element characterized to a good extent (Duilio et al., 2004). With a better understanding of induction mechanisms in cold-adaptation bacteria, more and more cold-stress induced genetic elements would be developed for efficient recombinant protein production.

**TABLE 1:** stress induction related genetic elements used for heterologous protein expression.

Genetic elements	Origin	Host	Regulation	Heterologous products	References
*CspA* (Promoter)	*E. coli*	*E. coli*	Low temperature	β-galactosidase, psychrophilic proteins	Vasina and Baneyx, 1996; Vasina et al., 1998; Bjerga and Williamson, 2015; Bjerga et al., 2016
*desaturase (des)* (Promoter)	*B. subtilis*	*B. subtilis*	Low temperature	β-galactosidase, xylanase, α-glucosidase	Thuy Le and Schumann, 2007; Janiyani and Ray, 2002
*pCnAFP* (promoter)	*Chaetoceros neogracile*	*Chlamydomonas reinhardtii*	Low temperature	Gaussia luciferase, mVenus fluorescent protein	Kim et al., 2020
*hutU* (Promoter)	*P. sychrotroph*	*P. sychrotroph*	Low temperature	urocanase	Janiyani and Ray, 2002
*PMtBL* (plasmid)	*P. haloplanktis*	*P. haloplanktis E. coli*	Low temperature	etpsychrophilic α-amylase	Duilio et al., 2004
*Rox1p* (transcriptional regulator)	*S. cerevisiae*	*S. cerevisiae*	Hypoxia	α-amylase	Liu et al., 2015; Huang et al., 2017
*ADH2* (Promoter)	*P. stipitis*	*P. stipitis, P. pastoris*	Hypoxia	green fluorescent protein, endo-1,4-xylanase, VHb	Passoth et al., 2003; Passoth and Hahn-Hägerdal, 2000; Gorgens et al., 2005; Passoth et al., 2003; Chien and Lee, 2005
*KlPDC1* (Promoter)	*K. lactis*	*K. lactis, S. cerevisiae, Z. bailii*	Hypoxia	β-galactosidase, interleukin 1β, laccase, glucoamylase, c-33 HCV	Camattari et al., 2007
*Hsp12* (Promoter)	*S. cerevisiae*	*S. cerevisiae*	multiple stress	green fluorescent protein	Karreman and Lindsey, 2005

For the translation and post-translational modification of proteins, temperature is also a crucial factor since the ribosome and associated proteins are all very sensitive to low temperature (Thomas et al., 2001). Intracellularly, the low temperature enables a slow-rate synthesis of foreign proteins, so that the nascent peptide chains may be allowed more time to fold in a proper way. Meanwhile, the misfolded or aggregated proteins are more sensitive to the proteolytic enzymes under low cultivation temperature, which allows properly folded protein to be secreted and misfolded protein to be degraded (Lee et al., 1990; Li et al., 2001). Zhong et al. found that high temperature would prolong the accumulation process of the nascent proteins in the endoplasmic reticulum (ER), which led to the overload of ER and cell death eventually. And that’s the reason why lower temperature is beneficial for cell viability and the fold capacity of ER (Zhong et al., 2014). Vera et al. (2007) demonstrated that the low temperature help with the conformational status of both soluble and insoluble polypeptides, meanwhile, it also affected the ratio of the proteins between soluble and insoluble state. Groot et al. suggested there was an inverse correlation between stability and activity of the inclusion bodies and the cultivation temperature in *E. coli.* The lower growth temperature was employed, the higher ratio of the right conformations in inclusion bodies was observed (de Groot and Ventura, 2006). Furthermore, a low cultivation temperature can result in a reduced extracellular proteolysis (Jahic et al., 2003; Torija et al., 2003; Zepeda et al., 2018).

### Osmotic Shock

Osmolality is caused by fluctuations of extracellular solute concentrations. Osmotically active compounds may either be ionic or uncharged. Because of the high cell density and high product concentrations, a high osmolality is always unavoidable (Mattanovich et al., 2004; Dragosits et al., 2011; Yang et al., 2014). High osmolality may interfere with cellular water availability, ion homeostasis, and turgor pressure regulation directly (Dragosits et al., 2011). And there is evidence that the increased osmolality has a beneficial effect on recombinant protein expression in diverse host cells, especially the mammalian organism cells.

In order to maintain the intracellular osmotic pressure, bacteria will response to high osmolality by transporting and synthesizing small organic osmolytes under a high osmolality condition. Osmolytes can be used as chemical chaperones and benefit protein stability by promoting protein refolding and disrupting protein aggregation (Diamant et al., 2003; Schlicke and Brakmann, 2005). Glycine betaine is one of the most common osmolytes in bacteria, whose interaction with the folded protein surface, but not the exposed protein backbone, plays an important role in regulating the free energy of the denatured state and changing the balance to improve the native state (Bolen and Baskakov, 2001; Felitsky et al., 2004). Under an osmotic stress condition, the existence of glycine betaine can even help with a compatible-solute-assisted periplasmic microenvironment, which is beneficial for recombinant protein production. As reported, an addition of 10 mM glycine betaine to *E. coli* under an osmotic stress condition help bacteria with a proper growth microenvironment and high concentrations of immunotoxins folded in native conformation (Barth et al., 2000). In Picaud’s research, the best production of heterologous amorpha-4,11-diene synthase and germacrene synthase in *E. coli* is achieved by adding 2.5 mM glycine betaine and 660 mM sorbitol to cultivation environment (Picaud et al., 2007). Reyes et al. employed osmotic shock to improve production and activity of *N*-acetylgalactosamine-6-sulfatase from human in *E. coli*. According to their research, osmotic stress not only increased the proper folding of recombinant protein at the cytoplasmic space, but also improved the secretion of target protein by accumulation of osmolytes which act as chemical chaperones (Reyes et al., 2017). Response to high osmotic stress, yeast cells will also accumulate osmolytes, especially glycerol, to maintain the intracellular osmolarity and achieve a more optimal state for recombinant protein production. It is reported that cultivating *P. pastoris* in a hypertonic medium first and then transferring it to the induction medium resulted in a dramatic increase in the production of a single-chain antibody (scFv) (Shi et al., 2003).

Except for the chemical chaperones, the osmolytes can also cause osmotic shock to microorganism, resulting in the overexpression of intrinsic chaperons. Such chaperons can help proteins with correct folding to the native state, simultaneously, refold the misfolded and aggregated proteins by forming a quality control system (de Marco et al., 2007). For example, in *E. coli*, the DnaK system and GroEL system are two quality systems controlling the protein aggregation and promoting the misfolded proteins refolding. Here, DnaK system works with ClpB to deal with aggregated proteins and solubilize them. And then, DnaK system cooperates with GroEL system to refold them to the native state (Mogk et al., 1999; Watanabe et al., 2000). Besides the DnaK and GroEL systems for protein folding, the *E. coli* also needs the DsbA/DsbC system for proper disulfide bond formation. DsbA is one of the strongest oxidases and will oxidize cysteine consecutively. While DsbC is a disulfide bond isomerase to shuffle the mis-matched disulfide bonds and produce its native folded state. The integration of the DsbC gene into the genome of a new *E. coli* protein expression strain led to the efficient synthesis of correctly folded multi-disulfide bonded proteins (Lobstein et al., 2012). Although various chaperone systems in *E. coli* including DnaK/DnaJ/GrpE (KJE), GroEL/GroES (ELS) and DsbA/DsbC are activated by the osmotic shock, a recent work focusing on the effect of individual chaperones on the increased activity of recombinant protein suggested that the positive effect is due to the global response rather than the function of a specific chaperones (Reyes et al., 2017).

Besides the enhanced protein folding and secretion facilitated by chaperones, many other intracellular activities such as ribosome biogenesis, cell wall organization, carbon metabolism and amino acid metabolism are affected by the increased osmolality (Dragosits et al., 2010). For instance, the glycerol-3-phosphate dehydrogenase GPD1 transcription in *S. cerevisiae* is induced and the glycerol pathway is thus activated. While the plasma membrane glycerol channel FPS1 is repressed, and the genes of ribosomal proteins and other proteins involved in translation were down-regulated. All the information obtained from the transcriptomics and proteomics can help us get a better understanding of the correlation between high osmotic stress and the ability of the host to produce recombinant proteins.

### Microgravity

As an extreme and unique environment, microgravity has significant effects on numerous microbial characteristics. Some researchers have proved that the microgravity condition is beneficial for microbial growth and their capacities to express recombinant proteins (Huang et al., 2012). Because it is difficult to attain the actual microgravity conditions in spaceflight due to the high cost and long duration of spaceflight, The specialized ground-based bioreactors were designed to simulate the weightlessness condition which was characterized by low sedimentation, low shear stress, and low turbulence (Huangfu et al., 2020). To achieve this, the equipment employed for providing microgravity should permit cell growth in suspension and minimize the fluid shear levels encountered by cells. Therefore, the designed bioreactors normally contain a cell culture vessel which rotates to make microbial cells not settle down but revolve around a horizontal axis and continuously fall through the fluid at 1 × g terminal velocity condition, leading to 1∼2 × 10^–2^ gravity environment. The discipline of suspension mechanical culture needs a systematic analysis of the relationship between mechanical culture conditions and biological effects, which has been reviewed extensively (Hammond and Hammond, 2001) and will not be covered here.

Qi et al. (2011b) explored the effect of simulated microgravity (SMG) on foreign protein production in *P. pastoris* and found that the production efficiency of recombinant β-glucuronidase in *P. pastoris* was enhanced by 1.5 to 2.2-fold under SMG at different rotary speeds when compared to normal gravity. Furthermore, a higher secretion efficiency (above 30% increase) was also achieved. More interestingly, the recombinant β-glucuronidase expressed under SMG showed a higher stability and catalytic efficiency than those of normal gravity though the exact mechanism is unknown. Xiang et al. (2010) investigated the effect of SMG on *E. coli* expressing recombinant β-glucuronidase, their results showed that both the cell dry weights and the production efficiency of the recombinant β-glucuronidase in SMG were higher than those in normal gravity (NG) control. The expression of endo-β-1,4-xylanase and β-glucuronidase under SMG condition is 2.81-fold and 2.43-fold higher than NG control in *P. pastoris*, respectively (Huangfu et al., 2015a). An *S*-adenosyl-L-methionine-producing *S. cerevisiae* strain H5M147 was cultured in spaceflight and the *S*-adenosyl-L-methionine yield was increased by 86.9% as compared to the control strain (Huang et al., 2012).

Some researchers analyze the changes of host cells at both transcriptomic and proteomic levels under the microgravity condition. The most significant results include the upregulation of ribosome/RNA polymerase genes and genes involved in energy metabolism and protein folding (Klaus, 1998; Demain and Fang, 2001; Johanson et al., 2002; Vukanti et al., 2008; Van Mulders et al., 2011; Kim et al., 2014; Huangfu et al., 2020). Therefore, the benefits of SMG on host cells can be summarized as follows. First of all, compared to NG, SMG shortened the lag phase of microorganisms and thus improved the cell density and exponential growth rate (Huangfu et al., 2015b). Secondly, according to proteomic analysis, some proteins or enzymes involved in oxidative stress response, methanol metabolism and carbon metabolism were significantly up-regulated under SMG (Qi et al., 2011a). Qi et al. demonstrated a significant upregulation of thiol peroxidase in *P. pastoris* under SMG, which was responsible for weakening the oxidative stress and regulating the transcription of various cellular proteins (Huangfu et al., 2015a). The upregulated genes involved in methanol metabolism could promote oxygen and methanol uptake, and enhance the transcription of the heterologous genes in *P. pastoris*. Besides, the high proteomic level of chaperones indicated that SMG condition would overload the endoplasmic reticulum (ER) folding machine, resulting in the aggregations and other response behaviors of nascent proteins. And the high transcriptional level of ribosome proteins demonstrated a promoted cellular protein synthesis machinery under SMG condition (Johanson et al., 2002; Qi et al., 2011a, b; Huangfu et al., 2015a). At last, the microorganism membrane has been changed to be connected more loosely under SMG, resulting in enhanced secretion of recombinant proteins (Qi et al., 2011b). Although the detailed mechanisms that allow recombinant microbial cells to response to the SMG environment are indistinct, the microgravity condition still shows great potential in adjusting microbial activity and ability of producing recombinant proteins.

### Hypoxia

There is an important interrelation between the production of recombinant proteins and hypoxia cultivation condition in yeast. Baumann et al. achieved a 2.5-fold increase in specific productivity of recombinant antibody under hypoxia condition in *P. pastoris*. In Baumann’s research, the amount of target heterologous protein Fab increased in *P. pastoris* (Baumann et al., 2008), when the oxygen concentration decreased from normoxic condition to hypoxic condition. According to the work by Garcia-Ortega et al., under the desired oxygen-limiting condition, a threefold increase of specific Fab production rate has been achieved in *P. pastoris* (Garcia-Ortega et al., 2017). Furthermore, the effect of anaerobic conditions on the production of recombinant proteins in *S. cerevisiae* has also been investigated. Rox1p is a transcriptional regulator which represses genes induced in hypoxia. ROX1 deletion led to a 100% increase in the recombinant fungal α-amylase yield as well as productivity (Liu et al., 2015). Huang et al. (2017) also confirmed that in the mutant strains of the yeast *S. cerevisiae* with a five-fold varying protein secretion capacity for a recombinant protein, several genes which are expressed under anaerobic/hypoxic conditions are significantly upregulated.

Similar to the cold shock inducible promoters, hypoxia-induced promoters such as P_ADH__2_, alcohol dehydrogenase gene promoter of *Pichia stipitis* (*P. stipitis*) and *KlPDC1* promoter of *Kluyveromyces lactis* (*K. lactis*) have also been employed for recombinant protein production. For instance, the promoter P_ADH__2_ was first employed in *P. stipitis* to express heterologous green fluorescent protein (Passoth et al., 2003) and endo-1,4-xylanase (Passoth and Hahn-Hägerdal, 2000; Gorgens et al., 2005). Afterward, the promoter P_ADH__2_ was applied in *P. pastoris* for the expression of bacterial hemoglobin (VHb). The production of VHb in *P. pastoris* at low O_2_ saturation was enhanced by 24-fold under the control of P_ADH__2_ when compared to that at 60% O_2_ saturation (Passoth et al., 2003; Chien and Lee, 2005). *KlPDC1* is the unique gene coding for pyruvate decarboxylase (PDC) in *Kluyveromyces lactis* (*K. lactis*) and its transcription is induced by hypoxic growth conditions. An expression system based on the *KlPDC1* promoter was developed and several heterologous proteins, differing in size, origin and localization were successfully expressed in *K. lactis* with a production ratio between four and more than 100 (Camattari et al., 2007). Moreover, the transferability of this system to related yeasts such as *S. cerevisiae* and *Zygosaccharomyces bailii* (*Z. bailii*) was also verified.

The possibility of increasing production of heterologous products by hypoxia or low temperature is very interesting from an applicative point of view, because hypoxic or cold conditions are easily and cheaply obtained in a bioreactor by reducing the air supply or adjusting the temperature and neither modulation of medium composition nor addition of inducers is required. Furthermore, besides the promoters in response to specific stress factors, some promoters can be induced by various stresses. For instance, the heat shock protein 12 (HSP12) promoter in *S. cerevisiae* can be induced by a variety of stresses, including high osmolarity, oxidative stress, heat shock and nutrient limitation. So the fusion of HSP12 promoter with green fluorescent protein (GFP) can be used to monitor the general stress status of yeast growing in an industrial application (Karreman and Lindsey, 2005; Table 1).

Besides the hypoxia induced promoters for direct transcription regulation, the hypoxia can also cause a global regulation to various intracellular activities, which can impact the heterologous protein expression indirectly. Herein, the physiology adaptation of *P. pastoris* and *S. cerevisiae* was analyzed to explore the reason why hypoxia can contribute to heterologous protein expression. The adaptation behaviors of yeast to restricted oxygen are involved in many important processes like respiration, carbon metabolism, lipid metabolism, amino acid metabolism and even vitamin metabolism (Wiebe et al., 2008; Baumann et al., 2010; Bendjilali et al., 2017). Under the oxygen-limiting condition, *P. pastoris* showed a strong transcriptional regulation of the carbon metabolism, including an increased transcriptional induction of glycolysis, the enhancement of the non-oxidative pentose phosphate pathways and the down-regulated TCA cycle (Carnicer et al., 2009, 2012; Baumann et al., 2010; Degreif et al., 2017), which may lead to the improvement of recombinant protein expression in *P. pastoris* (Garcia-Ortega et al., 2017). In addition, it was reported that yeast cells were able to regulate the composition of their membrane in response to restricted oxygen condition, leading to the changed composition of sterol and sphingolipid. The reduced ergosterol and increased sphingolipids would adjust the fluidity and permeability of the membrane and thus influence the secretion process of recombinant protein (Baumann et al., 2010; Adelantado et al., 2017; Degreif et al., 2017; Garcia-Ortega et al., 2017). Although hypoxia has been shown to positively affect heterologous protein production in both *P. pastoris* and *S. cerevisiae*, these two yeasts showed different adaptive responses to oxygen availability due to their different capacities to ferment glucose (Crabtree effect) (Baumann et al., 2011). The Crabtree effect defines whether a yeast can perform simultaneous respiration and fermentation under aerobic conditions at high growth rates. *S. cerevisiae* is Crabtree positive, while *P. pastoris* is Crabtree negative and thus more sensitive to the availability of oxygen. Therefore, a condition of oxygen limitation might be sensed by *P. pastoris* as more extreme, eliciting more notable impact on cellular processes related with protein secretion, the ergosterol biosynthesis, the central carbon metabolism and unfolded protein response. Therefore, a more significant increase in recombinant protein production might be achieved in *P. pastoris*. It is worth noting that both *P. pastoris* and *S. cerevisiae* have been successfully engineered to reverse their Crabtree phonotype for different use in biotechnology (Ata et al., 2018; Dai et al., 2018).

### Global Regulators Response to Environmental Stress

Environmental stresses usually evoke global stress responses in microorganism cells. These regulation processes are achieved by an important kind of molecules named global regulators. Hfq is a global regulator response to microgravity and is conserved in a wide range of microorganism species, regardless of prokaryotes and eukaryotes. The nature of Hfq is an RNA-binding protein which acts as a chaperone to interact with some RNA molecules, like mRNA, miRNA and siRNA (Valentin-Hansen et al., 2004; Wilson et al., 2007). When microgravity or other environmental stresses exist, the cell growth rate decreases, which in turn increases the intracellular level of Hfq. Hfq is significant to control the stability of mRNA and protect the RNAs from cleavage by RNase E, therefore facilitating recombinant protein production. In certain cases, one global regulator will be activated by multiple environmental stresses, such as stress-activated protein kinase (SAPK) Hog1, responsible for multiple resistant behaviors of *Candida albicans (C. albicans)*. Enjalbert et al. (2006) demonstrated that the inactivation of Hog1 would dramatically weaken the transcriptional responses of *C. albicans* to high osmotic stress, which showed the importance of Hog1 in the regulation of crucial stress response genes. The researches on global regulators are giving us guidance to understand the global response behaviors of microorganism cells under the stressful conditions.

## Omics Techniques Drive the Stress-Responsive Research for Construction of Efficient Microbial Cell Factory

**FIGURE 2:**
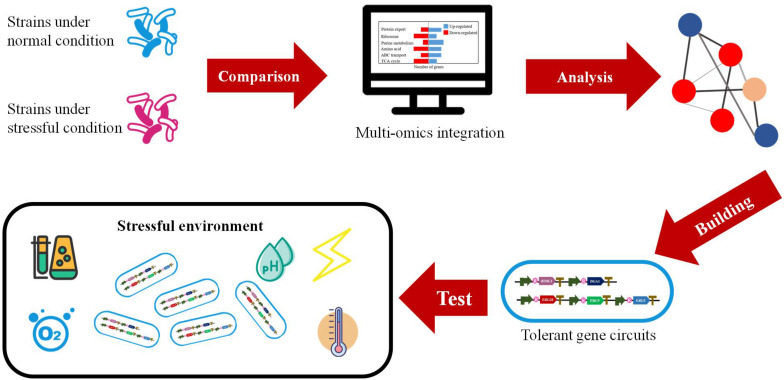
The construction of high-efficiency cell factories designed by multi-omics.

The development of omics-based analysis has enabled quantitative insights into the transcription and translation of all the genes and even the detailed metabolic networks under different conditions. The tool of omics has been frequently utilized to discover the impacts of environmental stress on metabolic activities of host cells, which could facilitate the improvement of recombinant protein production. Undoubtedly, omics technologies bring new opportunities for us to construct high-efficiency cell factories for recombinant proteins and other valuable products (Becker and Wittmann, 2018; Figure 2).

Proteomic represents a track record on expression level of proteins and interactions between protein molecules to investigate the effect of environmental stress and related genetic regulations in microorganism (Haussmann and Poetsch, 2012; Shen et al., 2016). According to proteome data mining and verification experiment, Xiao et al. (2018) studied the interaction networks of transcription factors and identified two novel transcription factors, Mig1 and Srb2, whose functions are involved in improving thermal resistance of *S. cerevisiae*. A recent research revealed the mechanism causing enhanced heterologous β-glucuronidase production in *E. coli* under simulated microgravity by multi-omics. Its transcriptome and proteomic analysis demonstrated that ribosome function and carbon metabolism were the two main points affecting the differential expression genes response to microgravity (Huangfu et al., 2020). As discussed above, hypoxia conditions can benefit recombinant protein production in yeasts. A quantitative metabolomics study of recombinant *P. pastoris* revealed that the pools of intracellular amino acids were decreased upon oxygen limitation, which was significantly influenced by the expression of recombinant protein (Carnicer et al., 2012). Carneiro et al. analyzed the metabolic fingerprints from *E. coli* W3110 and the Delta relA mutant strain under different cultivation conditions and observed a reduction of amino acid precursors and energy metabolism. Furthermore, various environmental stresses and overexpression of recombinant proteins usually exert a negative impact on cell growth and cause stress response. In a recombinant antibody producing *S. cerevisiae*, the antibody production reduced its maximum growth rate and prolonged its lag period. The metabolomics data showed prominent changes in metabolites involved in amino acids and redox metabolism, while the metabolic burden was embodied by the activation of the UPR in the recombinant strains, which can also influence the recombinant protein production (de Ruijter et al., 2018).

Muti-omics can also be employed to engineer the microorganism cell factories with more resistance and robustness. Xu et al. developed an automated high-throughput platform, based on which, more robust yeast cells with multilevel defense system could be screened for better industrial applications. According to the transcriptomes, they identified target genes benefiting for yeast robustness which are related to carbon metabolism, amino acid metabolism, glycolysis, HSPs and redox proteins (Xu et al., 2020). The analysis at the omics level actually opens a door for us to optimize the industrial strains and the production process for a desired yield of various products. There are many elaborated studies demonstrating the value of integrating multi-omics data for deciphering microbial metabolism and regulation, which will provide us a better understanding of the different layers of cellular control and most importantly, will bring a revolutionary change in the industrial biosynthesis mode in the future.

## Discussion

**FIGURE 3:**
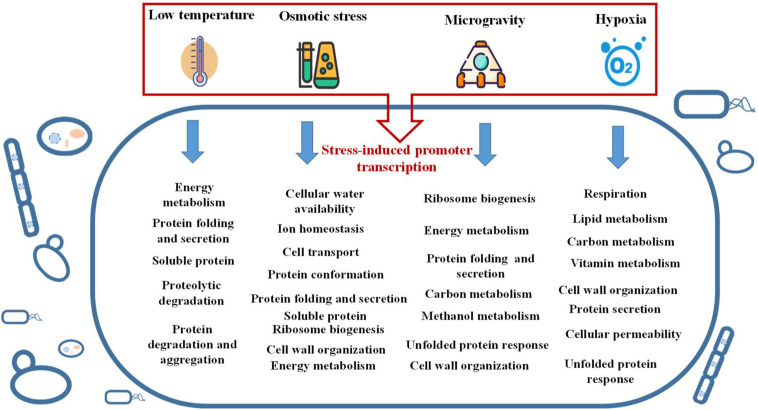
The cell activities interfered by different environmental stresses.

Different from conventional cultivation methods, here, we highlight the special or stressful conditions that have regulation effects on heterologous protein expression, and discuss the potential mechanisms behind them. In summary, the stress factors including low temperature, hypoxia, microgravity and high osmolality all play a positive effect on recombinant protein expression in microorganism hosts. The positive effects can be attributed to mainly two reasons. Firstly, the specific stress induced promoters can be employed directly to promote the target protein expression. Secondly, the stresses can cause a global regulation and general stress responses to the host cells and produce an indirect impact on recombinant protein expression. Such environmental stress responses are mostly characterized by a transition to a new level of carbon metabolism, transcriptional regulation, protein folding and secretion, amino acid metabolism, lipid metabolism and other important processes to prepare the cells to adapt to new stressful environment (Figure 3). It is of great concern that the adaption mechanism of recombinant hosts to environmental stress usually leads to an increased foreign protein production.

In addition, it is of great interest to study the stress responses of microorganism cells under individual or complex conditions mentioned above, especially at a systematic multi-omics level. There is an obvious need for such detailed models, which will not only benefit the rational optimization of recombinant protein production, but also provide new insights into host cells about their adaption mechanisms to environmental stress. It is worth noting that, environmental stress induction represents an optimal solution to activate the target protein production withoutaddition of any inducer molecules, which is readily attainable and represents a cost-effective way for industrial production.

## Author Contributions

XC: original draft preparation. CL: manuscript reviewing and editing. HL: project administration and final manuscript supervision. All authors contributed to the article and approved the submitted version.

## Conflict of Interest

The authors declare that the research was conducted in the absence of any commercial or financial relationships that could be construed as a potential conflict of interest.
